# An Ecosystem Approach to Developing and Implementing a Cocreated Bachelor’s Degree in Digital Health and Biomedical Innovation

**DOI:** 10.2196/63903

**Published:** 2025-09-23

**Authors:** Patrícia Alves, Elisio Costa, Altamiro Costa-Pereira, Inês Falcão-Pires, João Fonseca, Adelino Leite-Moreira, Bernardo Sousa-Pinto, Nuno Vale

**Affiliations:** 1 MEDCIDS–Department of Community Medicine, Information and Health Decision Sciences, Faculty of Medicine, University of Porto, Rua Dr. Plácido da Costa, Porto, 4200-450, Portugal, 351 225513622; 2 RISE-Health, Porto, Portugal; 3 Centre for Research and Intervention in Education, Porto, Portugal; 4 Department of Biological Sciences, Faculty of Pharmacy, University of Porto, Porto, Portugal; 5 Faculty of Medicine, University of Porto, Porto, Portugal; 6 Surgery and Physiology Department, Faculty of Medicine, University of Porto, Porto, Portugal

**Keywords:** digital health, biomedical innovation, curriculum design, co-creation, health professionals’ education, ecosystem-centered: biomedical, education, health care, graduates, teaching, learning, digital transformation, health information system, health education, information system, health management, project management, ecosystem

## Abstract

This paper aims to describe the cocreation and development processes of an educational ecosystem-centered Bachelor’s degree in Digital Health and Biomedical Innovation (SauD InoB). This program is shaped by a multidisciplinary, intersectoral, and collaborative framework, involving more than 60 organizations in teaching activities, internship supervision, or hosting, most of which collaborated in needs assessment, curriculum development, and public promotion of the degree. In the context of health care digital transformation, this comprehensive Bachelor’s degree will respond to unmet demands of the labor market by training students with technological, research, and management skills, as well as with basic clinical and biomedical concepts. Graduates will become transdisciplinary, creative professionals capable of understanding and integrating different “languages,” reasoning, clinical processes, and scenarios.

## Introduction

Biomedical innovation may be defined as the process of creation and application of scientific and technological knowledge to improve health care and promote health and well-being [1]. It includes developing therapeutic and diagnostic health technologies, biotechnology, precision medicine, drug discovery, or health care digitalization [2], which may encompass developing and using technologies such as health information systems, artificial intelligence, and advanced health data analytics [3,4].

To address global health challenges, imposed by aging demographics and sustainability of health and care systems, biomedical innovation is expected to integrate digital health [1], which plays an increasingly important role in the organization and management of health systems and institutions, health research, and health interventions [5]. However, biomedical innovation and digital transformation processes are frequently long, costly, risky, and challenging [2], involving changes in management dynamics [6,7] of complex project ecologies in which knowledge creation, flow, and outputs depend on the communication between multidisciplinary areas and sectors of activity [1]. Such a demanding context is further rendered more complex by management challenges, such as interoperability problems, data security concerns, ethical and legal aspects, low digital and scientific literacy among patients [6], resistance to change [7], or a recognizably insufficient health professionals’ workforce, simultaneously trained in digital health and biomedical innovation areas [8-10].

Overcoming these challenges and promoting successful change dynamics depends on multiple factors, namely a bold vision shared with different actors, engagement with the society through the involvement of various stakeholders, infrastructures, or the involvement of professionals with technical and scientific expertise, capable of adjusting the changes of management processes to contextual and cultural specificities [6] and to create synergies between different disciplinary areas and sectors of activity.

Identifying the unmet need for these professionals led to the creation of an innovative and comprehensive Bachelor’s degree in Digital Health and Biomedical Innovation (SauD InoB) at the University of Porto, one of the largest Portuguese universities.

In Portugal, the BSc degree leads to the degree of “licenciado,” corresponding to the level 6 of the National Qualifications Framework and European Qualifications Framework. A BSc degree may range from 180 to 240 European Credit Transfer and Accumulation System credits (ECTS) and last from 6 to 8 curricular semesters [11].

The BSc degree was structured according to the requirements and milestones of the Portuguese Agency for Assessment and Accreditation of Higher Education (A3ES) and grounded on the first steps of Kern’s approach to curriculum development: (1) identification and characterization of a problem, (2) identification of training needs, (3) definition of goals and objectives, and (4) definition of educational strategies [12]. This process comprised innovative aspects, engaging from the outset on a comprehensive and collaborative educational ecosystem–centered approach focused on the enhancement of students’ experiences and outcomes and on the strengthening of their contributions to society through the development of creativity and innovation capacity [13].

The BSc degree was created within an educational ecosystem, involving academic, research, health care, business, and society (eg, patients’ associations). An educational ecosystem refers to the interconnected network of factors and processes that influence students’ learning and development. Just like a natural ecosystem where different elements, such as air, water, plants, and animals, interact to create a balanced environment, an educational ecosystem consists of various components that work together to shape the learning experience [14-16]. According to Bronfenbrenner’s Ecological Systems Theory, learning and development are influenced by factors, processes, and actors situated in different contexts, some of them more directly involved in the learning process, such as family, teachers, higher education institutions, or internship sites (microsystem), others situated in a broader context, such as the course regulations (macrosystem), or the economic situation and cultural values that may influence learning (exosystem). Furthermore, learning may also be influenced by the interactions between contexts, for example, between the universities and industry (mesosystem) and by the dimension of time, for instance, by the effects of the technological development (chronosystem) [17].

Like natural ecosystems, educational ecosystems involve disturbances that may challenge their balance and require resilience, flexibility, and responsiveness to the needs and characteristics of different contexts and actors [14]. The concept of ecological university seems to entail this notion of disturbance and adaptive management. With the development of knowledge societies, higher education institutions no longer occupy a hegemonic position in the creation and legitimization of knowledge, and the actions of the individual and collective knowledge creators, users, legitimizers, and beneficiaries are informed by a widening diversity of perspectives and voices (eg, companies also have their own laboratories). In this context, higher education institutions must be able to interact with other contexts or ecological zones (eg, industry, patient associations, and government) with different rhythms, interests, epistemologies, and perspectives [18-20], but also to cross boundaries and build bridges between those contexts, not only to maintain their legitimacy, but also to be able to contribute to the health and functionality of the ecosystem [14].

Some authors concluded that the involvement of diverse stakeholders may promote an alignment with the needs of patients [21] or with the specific needs of diverse populations [22] and foster creativity, entrepreneurship, and innovation [13,23]. Curriculum development processes in transdisciplinary areas involving multidisciplinary and multisectoral teams tend to be complex processes in which tensions can arise, for example, related to different interests and rules [15]. Nevertheless, research on ecosystem-based curriculum development is still scarce, leaving the actors involved in these processes without a consistent theoretical basis to support their practices and decisions.

This viewpoint aims to contribute to the knowledge about curriculum development by describing the educational ecosystem–centered, multidisciplinary, and multisectoral cocreation and development process of the SauD Inob BSc degree, from needs assessment and curriculum design to the public promotion of the degree, aiming to attract its first group of students and the results of the first edition of the degree application.

## The Educational Ecosystem

The creation of this BSc involved 3 faculties within 1 university, the collaboration of 3 polytechnic institutions, and partnerships with more than 60 organizations, including hospitals and other health care providers, pharmaceutical and health technologies companies, research and governmental institutions, and patients’ associations. Cocreation processes involved more than 130 contributors, including faculty, researchers, master’s and PhD students and graduates in health, health informatics and data science, professionals working in diverse functional areas and sectors of activity, and managers and policy makers. The recruitment of students and alumni was strategically carried out in relevant master’s and doctoral programs, with some focus on digital health and biomedical innovation (medical informatics, clinical and health services research, and health data science), ensuring a highly relevant and involved participant base. Besides contributing to the BSc degree through involvement in teaching and internship supervision or hosting internships, most partners and collaborators were also integrated into needs assessment and curriculum development.

A list of the partners involved in the course is listed in [24].

## The Need for a BSc Degree in Digital Health and Biomedical Innovation

Needs assessment was based on a narrative literature review, document analysis, and meetings with the stakeholders integrated into the educational ecosystem. Individual meetings with partner representatives were carried out to discuss their perspectives on the interplay between labor market needs and the curriculum of SauD InoB and to establish a collaborative commitment. The curriculum was also presented and discussed in the first meeting of partners of the BSc degree in Digital Health and Biomedical Innovation in September 2023.

A literature review revealed a compelling but still unmet need to develop digital health and biomedical innovation skills and knowledge among the different professional groups involved in health care provision [8-10,25]. This need has been enhanced by a remarkable acceleration of the development of information and communication technologies, with repercussions for the adoption of electronic health records, the development of apps for monitoring chronic diseases, or advances in data analysis [3,4]. This unmet need motivated the introduction of digital health and biomedical innovation contents in the curricula of health and information technologies degree-awarding courses, the development of continuous education courses in biomedical and health informatics [26], and the creation of a few degree-awarding courses, in digital health and biomedical innovation [27-31], such as the BSc in Digital Health (Politechnic Institute of Porto, Portugal), the BSc in Digital Technologies and Health (University Institute of Lisbon, Portugal) [27], the Master’s Degree in Digital Health (Deggendorf Institute of Technology, Germany) [28], or the Master’s Degree in Digitalization in the Health Sector (University of Oslo, Norway) [31]. Nevertheless, the stakeholders involved in SauD InoB creation considered that the training offered in these areas is still insufficient to overcome the challenges of this fast-paced development context, namely in the North of Portugal.

The stakeholders identified the need to develop new professional profiles of transdisciplinary health care professionals who may establish bridges between different disciplinary or professional areas, academic and research cultures, knowledge, languages, and epistemologies. Graduates from SauD InoB are expected to be able to hold careers in (1) data analytics and artificial intelligence in health, (2) biomedical and clinical research, (3) health information systems and telemedicine, and (4) health consulting and management, innovation management, and other health-related areas. The labor market for these professionals may include digital technology and health informatics, pharmaceutical, medical device, and biotechnology companies, hospitals and health care institutions, research and development organizations (biomedical and clinical research laboratories, academia, research and development service companies, and consulting firms), and health policy and regulatory organizations.

## Aims and Learning Outcomes of SauD InoB

SauD InoB aims at training health professionals capable of (1) understanding concepts and “languages” in human biology, clinical medicine, and health care services and (2) mastering skills in health information systems, programming, data science, and research methodology. Therefore, these professionals will be able to identify, develop, and solve the main problems associated with health care services and with their digital transformation, offering them practical solutions that bridge different fields.

Upon completion of the study cycle, graduates should be able to apply critical knowledge about (1) human biology; (2) the pathophysiological basis of health and disease, different types of diseases, diagnostic tests, and therapeutic interventions; (3) clinical language and reasoning; (4) mathematics and statistics; (5) informatics, computing, and programming; (6) organization, pathways, and processes of health care provision in multiple contexts; (7) health care management and innovation; and (8) ethical, regulatory and security, data privacy in digital health, and health innovation processes. They should also be able to participate in the development, design and implementation, or management of databases, health care research and innovation projects, digital health systems and strategies, and apply data science approaches in health (processing, statistical analysis, and presentation of data).

SauD InoB goes beyond developing specific digital health and biomedical innovation competencies. It aims to create a new professional identity, which is defined as the individual sense of identification with a (new) profession and a sense of belonging to a community of professionals sharing specific knowledge, competencies, values, activities, and norms [32]. This is a distinctive characteristic of SauD InoB derived from being a BSc and not a subsequent degree, in which students’ professional identities are more likely to be influenced by the roles and competencies that characterize the professional or disciplinary area of their initial academic training [32].

Further information on the objectives, expected learning outcomes, and competencies of SauD InoB can be found on the course web page [33].

## Curriculum and Teaching and Learning Methods

SauD InoB comprises 6 academic semesters and 180 ECTS, integrating standardized and pluralized curricular components [34]. ECTS are units of learning based on learning outcomes and their associated workload and were defined to promote transparency and mobility within the European Higher Education Area.

Multimedia Appendix 1 lists the curricular structure of SauD InoB. The first 4 semesters (120 ECTS) constitute a common core, including a set of curricular units that all students must complete.

**Figure 1.**
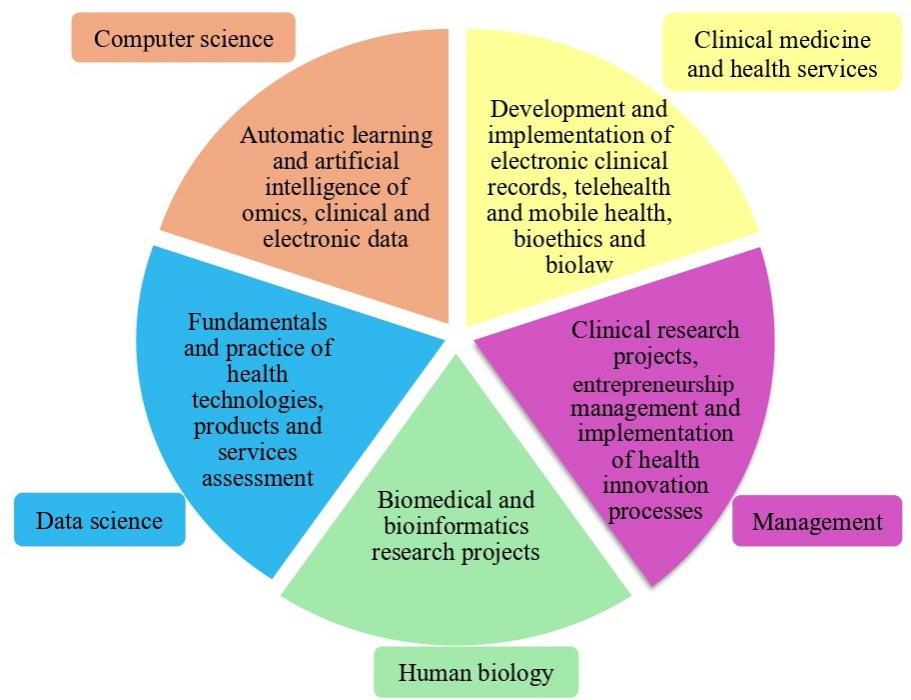
Fundamental areas of SauD InoB and main topics addressed by each area.

These 120 ECTS are divided into the areas of Computer Science (29 ECTS), Data Science (26 ECTS), Human Biology (26 ECTS), Clinical Medicine and Health Services (25 ECTS), and Management (11 ECTS). Elective units correspond to 3 more ECTS. Figure 1 shows the fundamental areas of SauD InoB.

In the last 2 semesters (60 ECTS), students must choose 1 of 4 branches, each of them with a stronger focus in 1 of the fundamental areas of SauD InoB: (1) Data Analytics and Artificial Intelligence is more focused on Data Science, (2) Health Information Systems and Telemedicine is more focused on Computer Science, (3) Clinical Research and Health Innovation Management is more focused on Clinical Medicine and Health Services, and (4) Biomedical Research and Bioinformatics is more focused on topics related to Human Biology. The creation of these branches was aligned not only with the fundamental scientific areas of the course but also with the professional profiles defined earlier by the educational ecosystem stakeholders. Each branch comprises 3 internship curricular units in the first semester and a project curricular unit in the second semester, in addition to a set of core and elective curricular units related to the subject.

Thus, the course enables an early differentiation in these profiles while providing introductory training in the different areas of knowledge, even in the third year, which confers a valuable competitive advantage for the labor market and responds to a pressing need in the health sector for professionals who have an overview of the sector and can build bridges between different professions and areas of knowledge.

The BSc includes theoretical, theoretical-practical, laboratory practice, and seminar classes, in which active teaching-learning methods, which promote interactions, will be privileged (eg, discussion of fictitious or real case studies, problem- or project-based learning, and flipped classroom). All branches include a project curricular unit in collaboration with one of the partners. Professional practice will be promoted through short-term observation and immersion activities and 3 internships in different areas in the third year. Despite its transdisciplinary approach, as health care processes are the central focus of SauD InoB, all branches include an internship in clinical medicine and health services.

Horizontal and vertical articulations will be promoted, for example, through use cases discussion, assignments involving various curricular units, simulation of patients’ pathway in the health care services, and assignments fostering progressively complex, transdisciplinary, and critical approaches.

Teaching and learning activities will be supported by education technologies (eg, e-learning platforms and medical simulation devices).

## Preliminary Assessment, Accreditation, and Funding of Saud InoB

The creation of new cycles of studies in Portugal requires a preliminary assessment and accreditation process [35,36], comprising the digital submission of a detailed proposal. After a preliminary analysis of compliance with national regulations and quality guidelines, the proposal is assessed by an external assessment commission. Besides assessing the written proposal, the commission conducts site visits and consultations and issues an evaluation report, which is the basis for the accreditation decision. More detailed information about accreditation can be found on the A3ES website [35].

The process of cocreating this BSc degree began in August 2021 and entailed the elaboration of a brief and an extended creation and accreditation proposal. The proposals were approved by the Pedagogical and Scientific Councils of the faculties involved in the cycle of studies and by the dean and senate of the university and were submitted to A3ES approval in February 2022.

In March 2023, the A3ES External Assessment Commission, including 3 external experts, issued a preliminary assessment report, which configured a peer review process comprising advice on the curriculum design and other suggestions for optimizing the BSc degree (eg, changes in the name of the BSc degree and a decrease in the number of branches, requiring changes to the curricular structure and to the syllabi of some curricular units). The commission included an international bioinformatician with expertise in genome analysis, systems biology, and computational tools for biomedical research; a professor of pharmacology and medical doctor with a strong background in biomedical sciences and medical education; and a digital health and health care management expert, with leadership experience in eHealth systems and medical informatics.

The task force incorporated most of the suggestions in a final proposal, and in May of 2023, SauD InoB was approved. The conclusions of the final assessment report asserted that “this training offer is not only pertinent and very innovative, but also of the highest quality and demand. The professionals it will train will make an unrivaled contribution to biomedical innovation structuring” [37].

The assessment reports can be found at the A3ES website. More information about the composition of the External Assessment Commission can be found in its final report [38].

The first edition of the cycle of studies was held in the 2024-2025 school year.

This cycle of studies was also included in the application of the University of Porto to the funds of Portugal’s Recovery and Resilience Plan, a plan integrated into NextGenerationEU [39], aiming to restore sustained economic growth in the postpandemic period and to respond to the challenges of the dual climate and digital transition [40].

## Public Promotion of SauD InoB

Public promotion of SauD InoB emphasized that the new course aimed to promote health innovation in Portugal by creating professionals with skills suited to the current and future needs of the health care market. Prospective students were invited to deepen their knowledge of human biology, experience the daily work of health care services alongside doctors and health professionals, develop programming skills, and gain expertise in cutting-edge methods for analyzing biological or clinical data.

In structural terms, a plan was defined to publicize the new degree in May 2023 (soon after the course was approved by A3ES). Public promotion of SauD InoB included posts in social media, institutional newsletter and webpage, news media publications, direct marketing actions (eg, email, SMS, course, and flyer), distribution of course gifts, dissemination of the course in academic institutional events, education fairs and targeted events, activities and presentations in high schools, and a newsletter (SauD InoB News).

Multimedia Appendix 2 shows the strategic plan for the promotion of SauD InoB.

## Results of the Competition for Accessing the First Edition of SauD InoB

Admission to first cycles of studies in Portuguese public higher education institutions is limited to a maximum number of placements (numerus clausus) and requires an application through an annual competition held by the Directorate-General for Higher Education under the general regime, special conditions (eg, top-level athletes and permanent staff of the Portuguese Armed Forces), or special competitions (eg, applicants older than 23 years or holders of a previous BSc degree) [11]. The admission process under the general regime is based on a weighted average of internal high school and national exams (entry score), ranging between 0 and 200 points. Some courses may also require the fulfillment of specific prerequisites, such as physical or functional aptitude tests. In the first edition of SauD InoB, 40 vacancies were opened for the first phase: 35 for the general regime, one under special conditions (for top athletes), and 4 for a special competition (holders of a previous BSc degree).

SauD InoB received 284 applications for the general competition, of which 83 were first option (candidates may apply for up to 6 cycles of studies, indicating their order of preference). The entry scores of the admitted applicants ranged between 179 and 199, placing SauD InoB as the 27th (out of 1119) cycle of studies with the highest entry score in the country, and 36 of the admitted applicants chose the course as their first option.

Considering the special competition for holders of a previous BSc degree, SauD InoB received 14 applications and filled all 4 vacancies. Results from the competition under special conditions were not available when this article was written.

## Final Remarks and Future Work

This paper describes the cocreation and the development process of a BSc degree in Digital Health and Biomedical Innovation, intricately developed within an educational multidisciplinary and multisectoral ecosystem, aiming to train transdisciplinary, highly skilled, and creative professionals able to respond to the needs and challenges of the health care sector in the context of fast-paced innovation and digital transformation. Besides following the requirements and milestones for the creation and accreditation of cycles of studies as determined by A3ES, this creation process was based on Kern’s approach to curriculum development [12] and grounded on the concept of ecological university [18-20].

This ecosystem-centered cocreation of a bachelor’s degree enabled the team to learn a few lessons, which may be helpful for other researchers and curriculum developers:

1. The integration of diverse stakeholders in the cocreation process seems to have fostered the development of a more adapted curriculum, flexible and responsive to the needs of diverse employers and to the needs of the users of the services that will be delivered by the professionals trained by Saud InoB;
2. The results of the competition for accessing the first edition of SauD InoB and subsequent admission of students with high classifications confirmed the relevance of this BSc course. Furthermore, the vacancies under special conditions and the special competition may increase the diversity of students and contribute to the democratization of access to SauD InoB;
3. More research is needed on curriculum development methods.

Further work will be needed to monitor the implementation of SauD InoB and promote continuous adaptation of the education processes to the needs of students and other actors. This involves pursuing collaboration with its current partners, namely through regular partner meetings, widening community involvement by joining new partners, and designing tools and strategies to involve students (and, in the future, graduates) in the processes of continuous assessment and improvement of SauD InoB. Course assessment will be pursued through meetings with faculty and students’ representatives. The University of Porto also assesses all the courses through pedagogical questionnaires from the students. Furthermore, all the degree-awarding courses in Portugal are periodically assessed by A3ES.

This work may contribute to the knowledge about processes involved in creating new cycles of studies and informing academics and other stakeholders on the practices involved in such processes. Moreover, and even if the first edition of SauD InoB is still taking its first steps, this cocreation process and educational ecosystem–centered approach may be already considered a successful experience of productive interactions [41], which questions (even if not in an uncritical way) [18] a discourse of higher education in crisis [42,43] and reflects higher education’s more than ever active role in promoting bridges between different actors who, in a knowledge society, join forces to promote the improvement of societies and the well-being of the population.

## Supplementary material

10.2196/63903 Multimedia Appendix 1 Curricular structure.MA1_.docx

10.2196/63903 Multimedia Appendix 2 Strategic plan for the promotion of the new SauD InoB degree at the Faculty of Medicine of the University of Porto (FMUP).63903-Multimedia-Appendix-2.docx

## Abbreviations

A3ES: Agency for Assessment and Accreditation of Higher Education
ECTS: European Credit Transfer and Accumulation System credits
InoB: Bachelor’s degree in Digital Health and Biomedical Innovation
